# Diagnostic Accuracy of Panoramic Radiography for Assessing Maxillary Posterior Root Protrusion into the Maxillary Sinus: A Cluster-Adjusted Prediction Model Using CBCT as Reference

**DOI:** 10.3390/diagnostics16162580

**Published:** 2026-08-15

**Authors:** Duygu Ölmez, Nursel Akkaya

**Affiliations:** 1 Tepebaşı Oral and Dental Health Hospital, Ankara 06560, Türkiye; duygudugencili@gmail.com; 2 Department of Dentomaxillofacial Radiology, Hacettepe University, Ankara 06100, Türkiye

**Keywords:** cone-beam computed tomography, maxillary sinus, panoramic radiography, tooth roots

## Abstract

**Background/Objectives**: The proximity of the maxillary sinus floor to the tooth apices is critical for dental procedures, so preoperative evaluation is crucial for preventing complications. This study aims to examine the accuracy of panoramic radiographs in determining the relationship between the maxillary sinus and teeth, in comparison with cone-beam computed tomography (CBCT), which serves as the reference standard, and to develop a cluster-adjusted clinical prediction model to assist clinicians in identifying patients who may benefit from CBCT imaging. **Methods**: A total of 3436 teeth were evaluated using CBCT and panoramic radiographs for the relationship between root tips and sinuses. The McNemar–Bowker test and the R program were used to assess how accurate panoramic radiographs are by comparing them to CBCT imaging. The performance metrics of panoramic radiography were calculated, and the generalized estimating equation (GEE) logistic regression model and the interactive risk calculator were developed. **Results**: The McNemar–Bowker test indicated a statistically significant systematic difference between the two modalities (χ^2^ = 190.01, *p* < 0.001); the overall three-category agreement was 82.2% (cluster-corrected 95% CI: 80.4–84.0%), corresponding to a dichotomized (protrusion vs. non-protrusion) accuracy of 87.6%. A cluster-corrected GEE logistic regression model, including panoramic classification, tooth type, age, and sex and developed and internally evaluated in this single-center cohort, predicted root protrusion identified using CBCT with high apparent discrimination (AUC = 0.948; cluster-based bootstrap 95% CI: 0.941–0.955, 1000 resamples; optimism-corrected AUC = 0.948) and satisfactory apparent calibration. **Conclusions**: This model, developed and evaluated internally within a single-center cohort, has the potential to serve as a clinical decision-support tool to help prioritize the need for CBCT in patients pre-evaluated with panoramic radiography. However, its performance reflects apparent, in-sample estimates, and external validation in independent populations is required before it should be considered for routine clinical use.

## 1. Introduction

The maxillary sinus, the largest of the paranasal sinuses, was first described as the “antrum of Highmore” by an English surgeon, Nathaniel Highmore (1613–1685) [1]. Located bilaterally in the mid-face and present from birth, the maxillary sinus takes on a pyramidal shape with growth and development, with its base formed by the lateral wall of the nasal cavity and its apex extending toward the zygomatic bone [2]. The maxillary sinus reaches its final size upon completion of tooth development [3]. The upper wall of the maxillary sinus is formed by the floor of the orbit and the lower wall by the alveolar process of the maxilla [2]. In pubertal dental radiographs, the maxillary sinus and the base of the nasal cavity appear at almost the same level. Generally showing symmetrical growth, the maxillary sinus may extend towards the alveolar process in the posterior maxillary region in older individuals, and its floor may be seen at a lower level than the base of the nasal cavity [3]. 

The proximity of the maxillary sinus floor to the posterior teeth is critical for oral diagnosis and treatment planning. This floor may extend between adjacent tooth roots, or the roots of the teeth may protrude into the maxillary sinus. Odontogenic infections of the maxillary posterior teeth may spread into the sinuses and lead to odontogenic sinusitis [1,4,5]. In addition, benign or malignant tumors triggered by odontogenic infections may spread more easily through the maxillary sinuses [6]. This topographic relationship can lead to various complications during dental procedures, such as the formation of an oroantral communication and displacement of root canal fillings [7] and root fragments into the sinus [8,9,10].

The most important risk is the perforation of the Schneiderian membrane during tooth extraction or surgical dental procedures. Because of their close relationship, sinus membrane perforations most frequently occur in the first molar region, and the incidence of perforation following the extraction of the first molar is 3.8% [11]. A perforation rate of 9.6% has been reported in apical surgical procedures applied to premolars and molars [12]. Small perforations in healthy tissue can close spontaneously during the normal healing process as the socket fills with a blood clot. However, an oroantral fistula with a diameter greater than 5 mm cannot close spontaneously [13]. If the oroantral communication that occurs after the extraction of a tooth associated with the maxillary sinus is not treated, it can turn into an oroantral fistula [14]. A thorough understanding of the relationship between tooth roots and adjacent anatomical structures may reduce the likelihood of procedural complications. Therefore, prior to performing surgical interventions on the maxillary posterior teeth, it is essential for the clinician to undertake an extensive examination of the region [15]. 

Panoramic radiography is an imaging method frequently preferred by dentists to evaluate the relationship between teeth and surrounding structures before a planned surgical procedure [16]. However, this method provides two-dimensional information about anatomical structures, and it is not possible to obtain cross-sectional information related to anatomical relationships. Additionally, the superimposition of anatomical structures in the image can also lead to the insufficient evaluation of the pertinent area. Image magnification is another limitation of the technique [17]. These limitations are not exclusive to the maxillary sinus region; comparable superimposition- and magnification-related diagnostic limitations of panoramic radiography have also been demonstrated in a different clinical context, the assessment of impacted canines [18], supporting the broader relevance of this limitation across panoramic radiography applications.

Three-dimensional imaging methods are used for presurgical evaluation in cases where panoramic radiography is insufficient. Cone-beam computed tomography (CBCT) is a commonly used, three-dimensional imaging method before dental procedures for visualizing the structures of the head and neck regions without superimposition, as it delivers a significantly lower radiation dose to the patient compared to medical CT scans [17,19]. Providing high-resolution images with small isotropic voxels, CBCT imaging ensures accurate measurements in all planes [19,20]. Although CBCT provides considerable benefits, it also involves higher radiation exposure and greater costs compared to conventional imaging methods, which restricts its use. Therefore, panoramic and periapical radiography remain indispensable methods for initial evaluations in routine clinical practice.

Several studies have compared panoramic radiography with CBCT to visualize the root–sinus relationship [21,22,23,24,25,26,27,28]. Some studies have indicated a high level of agreement regarding roots distant from the maxillary sinus floor when comparing panoramic and CBCT imaging [21,23,26,27,28]. Hassan [22] found a lower correlation value between CBCT and panoramic radiography compared to other studies [21,23,26,27,28]. Jung et al. [25] recommended that CBCT be prescribed in cases where a panoramic image reveals root protrusion into the sinus. Kirkham-Ali et al. [26] noted that prior studies had used small sample sizes and had limited age ranges, resulting in inaccurate representation of the entire population. They suggested that an evaluation involving larger samples and a broader age range would be beneficial. These studies have analyzed diagnostic accuracy using conventional statistical methods that treat each tooth as an independent observation, even though multiple teeth from the same patient are inherently correlated; this can underestimate standard errors and overstate precision [21,22,23,24,25,26,27,28]. Furthermore, to our knowledge, no prior study has translated panoramic classification, together with patient-level clinical variables, into an individualized, quantitative clinical prediction tool that clinicians can use at the point of care to decide whether CBCT is warranted for a specific patient. This represents an important gap that the present study addresses. 

In this study, we aimed to assess the diagnostic accuracy of panoramic radiography, a method frequently used in clinical practice, for evaluating the relationship between the maxillary sinus and root apices, using CBCT images as the reference standard and incorporating statistical methods that account for clustering effects to provide more robust and reliable results and to develop a cluster-adjusted clinical prediction model.

## 2. Materials and Methods

### 2.1. Sample Size Calculation

The sample size was primarily calculated based on the precision of prevalence estimates for the three-category classification, with adjustment for clustering due to multiple teeth being evaluated within the same individual. Using the normal approximation for a binomial proportion, the required number of observations was estimated according to the following formula:

(1) $$n=Z^{2}\times p(1-p)/d^{2}$$

where n represents the required sample size, Z is the standard normal deviation (with a 95% confidence interval (CI)), p indicates the predicted proportion, and d is the desired precision. Z = 1.96 and p = 0.38, based on the prevalence reported by Lopes et al. [21], and d = 0.05 was used. This calculation yielded an initial estimate of approximately 363 teeth, under the assumption of independent observations. Because multiple teeth were assessed per participant, clustering effects were incorporated using the following design effect formula:

(2) DEFF=1+(m−1)×ICC

where m represents the average cluster size and ICC represents the intraclass correlation coefficient. In this context, m = 7.2 represents the average number of teeth per patient, while ICC = 0.30 serves as a conservative estimate of the intraclass correlation coefficient based on previous studies [29,30]. This resulted in a DEFF of 2.86 and an adjusted minimum sample size of 1038 teeth (approximately 145 patients). The final recruitment target was set at 162 patients, corresponding to approximately 1166 teeth, to compensate for the anticipated 10% rate of non-evaluable observations. This calculated value represents the minimum required sample rather than a recruitment ceiling. As this was a retrospective study, all consecutive patients meeting the eligibility criteria within the pre-specified institutional records review period were included, which yielded a substantially larger final sample (484 patients, 3436 teeth) than the calculated minimum.

### 2.2. Eligibility Criteria

The inclusion criteria were as follows: patients at least 18 years old; diagnostically adequate radiographic quality; an imaging field covering the maxillary dental arch and maxillary sinuses; an interval of no more than 6 months between CBCT and panoramic radiography (to avoid tooth loss or periapical pathology that could displace the sinus floor); and no fracture line or pathology in the relevant area. In previous studies, the time interval between the two imaging modalities ranged from 6 months [27] to one year [24]. A six-month window was chosen a priori as a pragmatic upper bound for this retrospective radiographic comparison study, balancing the need for an adequate sample size against the risk of anatomical change over time; the exclusion criteria below (extraction, new periapical pathology, or root canal treatment between the two imaging dates) were specifically intended to mitigate residual risk of interval-related anatomical change within this window. The teeth included complete root formation, normal eruption position, normal apical region, and no root abnormalities. Teeth were excluded from the study if they were retained/impacted, had a periapical lesion, or had root canal treatment or if image artifacts/superimposition precluded reliable classification on panoramic radiographs.

### 2.3. Radiographic Protocol

In our study, panoramic radiographs were obtained using Orthophos XG 5 (Sirona Dental Company, Bensheim, Germany; 60–90 kVp, 3–16 mA, 14 s) or Veraview IC5 (Morita Corporation, Kyoto, Japan; 60–70 kVp, 1–7.5 mA, 5.5–10 s) panoramic radiography devices with imaging parameters suitable for the patient’s physical structure. CBCT images were obtained using the i-CAT Next Generation (Imaging Sciences International, Hatfield, PA, USA) device. The exposure parameters were a tube voltage of 120 kVp; tube current of 5 mA; FOV of 16 × (6–13) cm; voxel size of 0.2 mm; and exposure times of 14.7 and 26.9 s.

### 2.4. Radiographic Examinations

In this study, CBCT images were used to define the types of relationships between the maxillary sinus floor and the root apices as the reference standard. The accuracy of panoramic radiography, as the index test, in evaluating the root–sinus relationship was examined by comparing it with data obtained from CBCT scans. Before beginning the evaluations, the two observers performed a calibration exercise on a different dataset consisting of 30 CBCT scans and panoramic radiographs. 

The CBCT images were visualized using the I-CAT Vision software (version 1.9.3.14, Imaging Science International, Hatfield, PA, USA) and were jointly evaluated by two observers as a consensus radiographic standard. In cases where there was disagreement, the opinion of a third observer was sought. The reference standard was deliberately selected as a consensus interpretation with third-observer adjudication of disagreements, reflecting clinical evidence that two-observer consensus outperforms single-observer readings [31]. The aim of this methodology was to reduce the misclassification of the reference standard, although it comes at the cost of preventing an independent assessment of CBCT interobserver concordance. In cross-sectional, sagittal, and coronal views with a 1 mm slice thickness, the type of root–sinus relationship for each tooth was noted. For this purpose, in multi-rooted teeth, the classification was made according to the root with the shortest root–sinus distance. 

**Figure 1:**
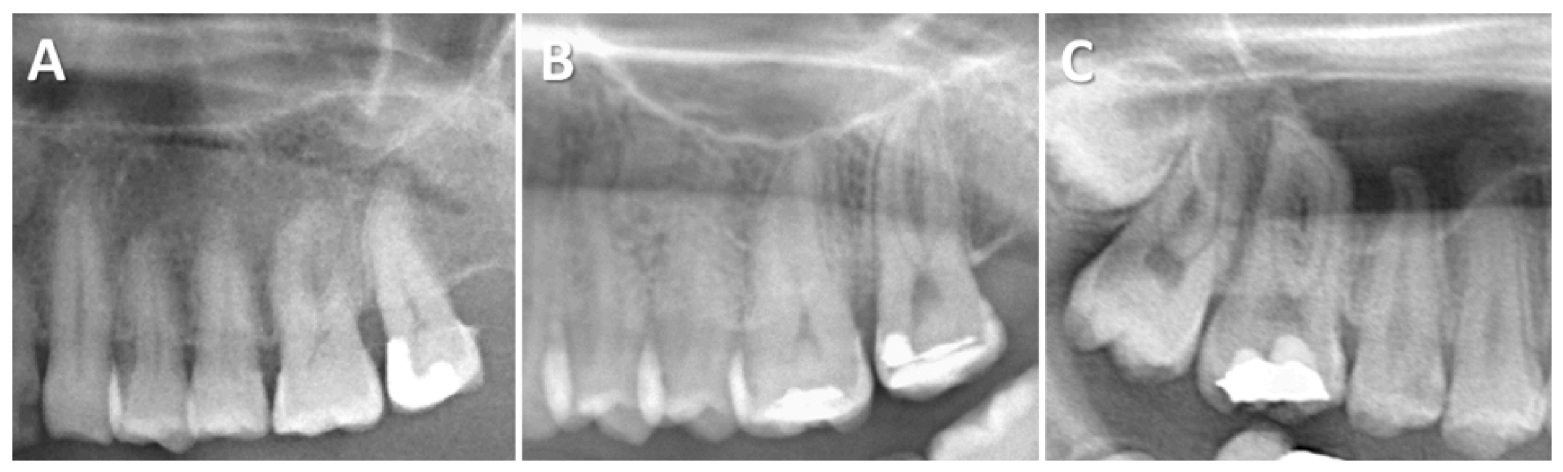
Cropped panoramic radiographs illustrating the relationship between the maxillary sinus floor and the roots. (**A**) Class 1: the root is distant from the maxillary sinus floor; (**B**) Class 2: the root is in contact with the sinus floor; (**C**) Class 3: the root is projected into the sinus cavity.

**Figure 2:**
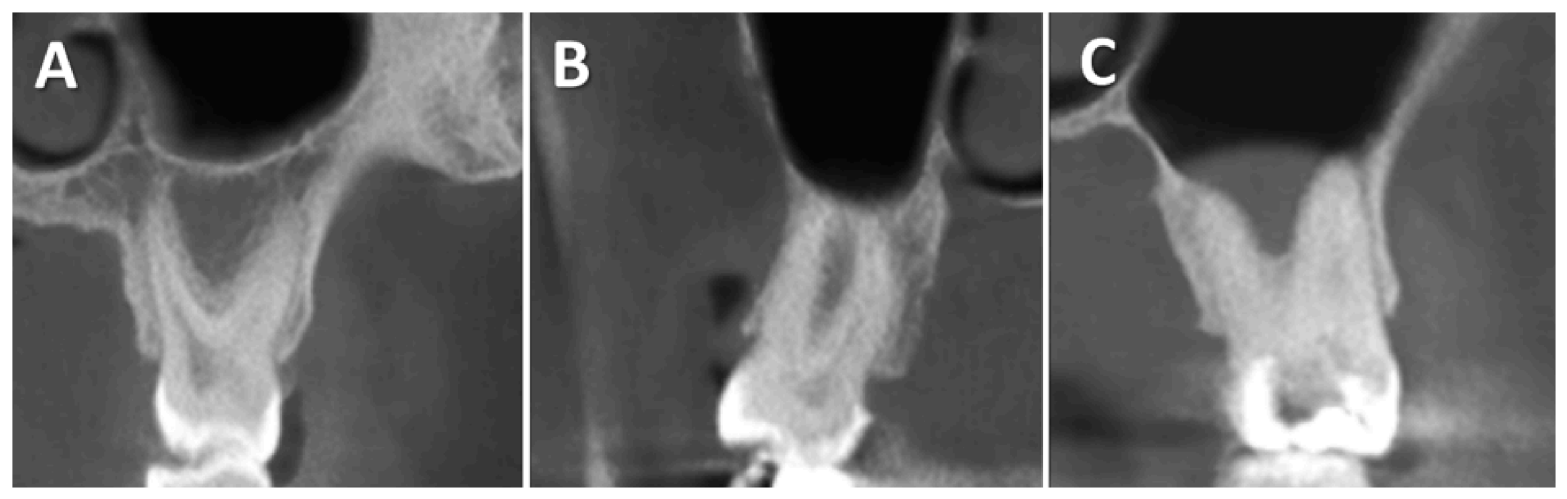
The relationship between the maxillary sinus and roots on coronal CBCT scans. (**A**–**C**) Same as Figure 1.

The evaluation of panoramic radiographs commenced two weeks after the completion of the CBCT assessment to reduce the likelihood of observers recalling their classifications. The same two observers evaluated both the CBCT reference standard and panoramic radiographs. Although the observers were not provided with the CBCT classification results at the time of panoramic scoring, this process constitutes a time-separated evaluation by the same individuals rather than a formal blinded evaluation by independent observers. Panoramic radiographs were assessed under suitable lighting conditions using the Windows 10 Photo Viewer (Microsoft, Redmond, WA, USA) on a 14.0 Lenovo C460 monitor (Lenovor, Pokfulam, Hong Kong), focusing on the relationship between the maxillary sinus floor and the roots of the maxillary canines, first and second premolars, and first and second molars independently by two observers. The relationship between the maxillary sinus floor and the root apex was evaluated using a modified scoring system adapted from the criteria established by Shahbazian et al. [23]. Root–sinus relationship classification: in Class 1, there is a distinct space between the root apex and the sinus floor; in Class 2, the root apex is in contact with the sinus floor; and in Class 3, the root apex is observed to project above the maxillary sinus floor during panoramic evaluation, while it protrudes into the sinus during CBCT evaluation (Figure 1 and Figure 2).

To test intra- and inter-observer agreement, 547 teeth in 65 randomly selected panoramic radiographs were independently evaluated by two observers in terms of the root–sinus relationship, with a minimum two-week interval between evaluations.

### 2.5. Statistical Analysis

The SPSS v. 23.0 (IBM SPSS Statistics for Windows; IBM Corp., Armonk, NY, USA) statistical program was used for data analysis, and the linear weighted kappa coefficient was used to evaluate intra- and inter-observer agreement. A descriptive analysis was conducted on the CBCT dataset, including the frequency distribution of relationship types according to tooth types. 

Considering CBCT as the reference standard, a contingency cross-table was created. The McNemar–Bowker test was used to assess systematic disagreement between the two imaging modalities, and the linear weighted kappa coefficient was calculated to assess the degree of agreement. Additionally, the overall agreement/correct classification rate between panoramic radiography and CBCT was calculated using the R program [32].

The sensitivity, specificity, positive (PPV) and negative (NPV) predictive values, and positive (LR+) and negative (LR−) likelihood ratios were calculated using CBCT as the reference standard. A *p*-value  <  0.05 indicated statistical significance. For diagnostic performance analysis, CBCT Class 3 (root protrusion into the sinus) was defined as the positive condition, and all other classes were considered negative.

Since multiple teeth were evaluated per patient, observations were not independent. The intraclass correlation coefficient (ICC) was calculated to quantify the degree of clustering. Given the high ICC (0.691) and the resulting DEFF (5.22), cluster-corrected 95% confidence intervals (CIs) were derived via patient-level bootstrap resampling rather than assuming independence across observations. The generalized estimating equations (GEEs) were applied to account for the clustering of multiple teeth within the same individual, thereby adjusting for intra-individual correlation. An exchangeable working correlation structure was assumed, and robust (sandwich) standard errors were used to obtain valid population-averaged estimates. This approach was preferred over conventional logistic regression, as ignoring clustering may lead to underestimated standard errors and inflated type I error rates. GEE logistic regression (exchangeable working correlation structure, patient as clustering unit) was used to identify independent predictors of panoramic misclassification while formally accounting for within-patient clustering. The dependent variable was correct classification (1 = correct, 0 = incorrect). Reference categories were as follows: ‘canine’ for tooth type, Class 1 for CBCT class, and female for sex.

A cluster-adjusted clinical prediction model was developed to improve the clinical prediction of root protrusion into the maxillary sinus, as identified using CBCT. The binary outcome variables were defined as ‘CBCT Class 3’ (roots protruding into the sinus) and ‘CBCT Classes 1–2’ (distant/contact). Panoramic radiographic classification, tooth type, age, and sex were included in the model as predictors (independent variables). These predictors were clinically prespecified based on established anatomical and epidemiological associations with root–sinus relationships reported in the prior literature [4], rather than selected through a data-driven stepwise procedure. For this prediction model, tooth type was pooled into three categories (canine, premolar, molar), in contrast to the five-category classification used in Table 1 to preserve statistical power and model parsimony for a tool intended for practical bedside use; a sensitivity analysis using the five-category classification is reported in the Supplementary Materials. Classification coding: Panoramic classification was modeled as a three-category ordinal variable: 1 = distance between the root and sinus; 2 = the root in contact with the sinus floor; and 3 = the root protruding into the sinus. The CBCT classification was originally recorded as 1–3; Classes 1 and 2 (distance/contact) were combined to form the reference category, and Class 3 (protrusion) was the event category, creating a binary outcome variable consistent with the diagnostic accuracy analysis above.

Model performance was evaluated for discrimination using the area under the curve (AUC), the 95% CI for AUC using patient-level cluster-based bootstrap resampling (1000 resamples), and calibration using observed–predicted probability comparisons based on decile, together with the apparent calibration slope and intercept. To assess overfitting, model optimism was additionally quantified using Harrell’s bootstrap optimism-correction procedure (1000 cluster-based resamples), in which the model was refit in each resample and evaluated both on the resample (apparent performance) and on the original data (test performance), with the mean difference subtracted from the apparent AUC to yield an optimism-corrected AUC. The incremental discrimination contributed by tooth type, age, and sex beyond panoramic classification alone was quantified by comparing the full model to a reduced model containing panoramic classification only (delta AUC), and a sensitivity analysis re-fitted the prediction model using the five-category tooth-type classification from Table 1 in place of the pooled molar/premolar/canine grouping. Clinical utility was further assessed using decision curve analysis (DCA), comparing the model’s net benefit against treat-all, treat-none, and two panoramic-classification-based heuristics across a range of threshold probabilities. All analyses were performed using Python version 3.12.3 (statsmodels GEE, Binomial family). The GEE logistic regression model, bootstrap confidence interval calculations, calibration assessment, and the interactive risk calculator were developed with the assistance of Claude Opus 4.8 (Anthropic PBC, San Francisco, CA, USA), a generative artificial intelligence tool. All statistical outputs, model coefficients, and calculator logic were independently reviewed and verified by the authors before use. 

## 3. Results

### 3.1. Diagnostic Accuracy of Panoramic Radiography

The results are reported in accordance with the STARD (Standards for Reporting Diagnostic Accuracy Studies) Statement [33]. The linear weighted kappa values for intra- and inter-observer reliability were 0.952 (95% CI 0.926–0.974) and 0.915 (95% CI 0.883–0.943), respectively, indicating excellent agreement within and between observers [34]. 

**Figure 3:**
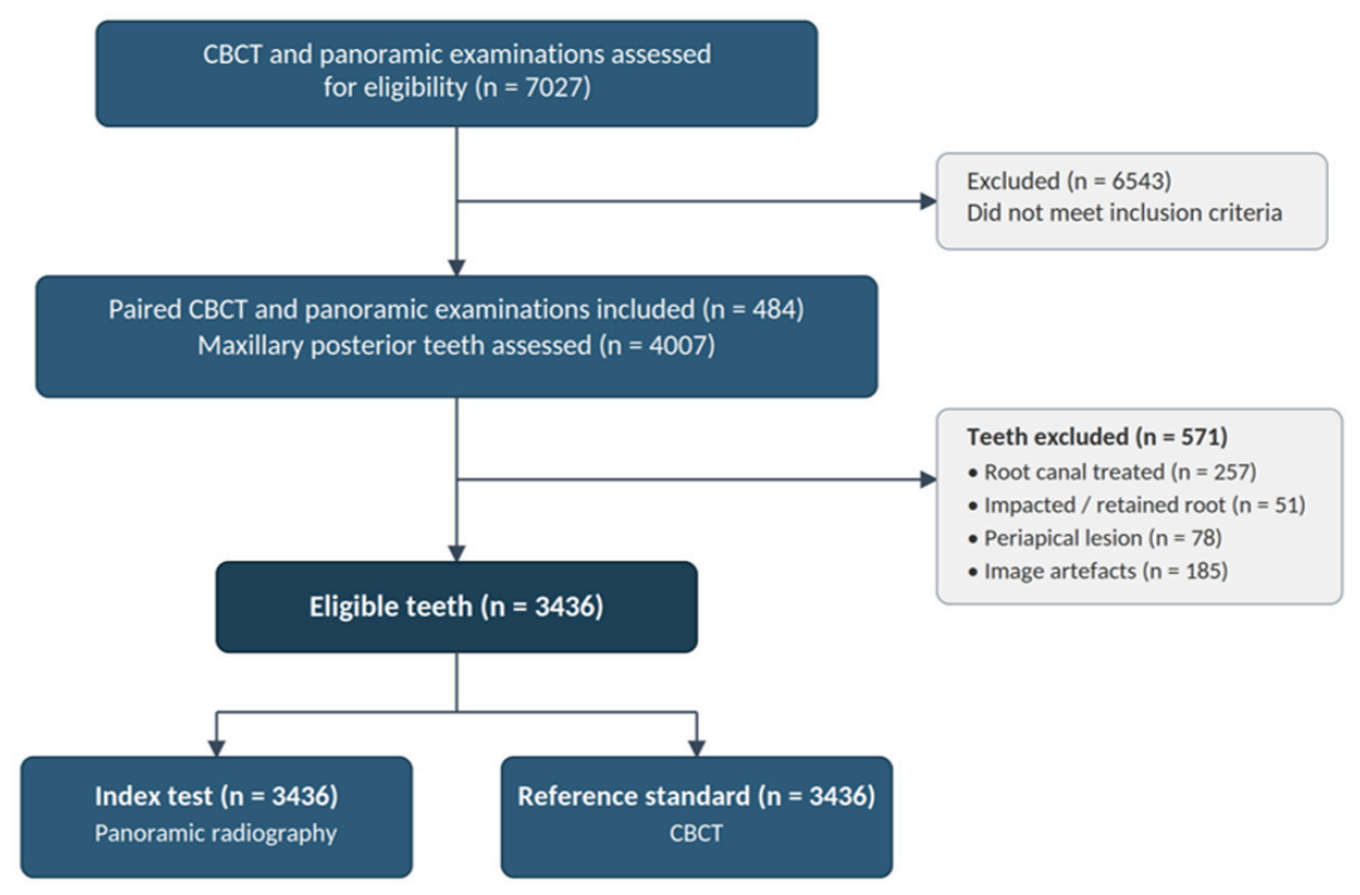
Workflow diagram detailing the selection of patients and teeth included in the study.

Radiographic records of 7027 patients were reviewed for suitability for the study. CBCT and panoramic radiographs of 484 patients were selected after applying inclusion and exclusion criteria. Examining 4007 maxillary posterior teeth from these 484 patients and excluding 571 teeth for various reasons, a final population of 3436 teeth was included in the study (Figure 3).

Of a total of 484 patients, 286 (59.1%) were females and 198 (40.9%) were males. The average age of the patients, whose age range was 18–84, was determined to be 38.11 ± 13.68. Of the 3436 teeth, 1724 (50.2%) were in the right maxillary region and 1712 (49.8%) were in the left maxillary region. There was no statistically significant difference for each tooth group between the right and left maxillary regions (*p* > 0.05). Therefore, without distinguishing between right and left, the data obtained from CBCT images were grouped according to tooth types and are presented in Table 1. According to the CBCT evaluation, 2125 (61.8%) teeth have a distance between the root and the sinus, while 773 (22.5%) teeth have roots that protrude into the sinus.

Table 2 presents the contingency table that illustrates the root–sinus relationship based on CBCT and panoramic imaging methods. The McNemar–Bowker test indicated a statistically significant systematic difference between the two modalities (χ^2^ = 190.01, *p* < 0.001). However, agreement analysis demonstrated substantial concordance between the methods (linear weighted κ = 0.762, cluster-corrected 95% CI: 0.746–0.779). The most prominent misclassification pattern is panoramic radiography categorizing Class 2 roots (in contact with the sinus floor) as Class 3 (protruding into the sinus) in 47.4% of cases (*n* = 255 teeth). Panoramic radiography tended to overestimate sinus involvement, particularly by misclassifying Class 2 cases as Class 3.

As a result of the analysis conducted using the R program based on the concordant classifications across the contingency table for each classification, the overall agreement/correct classification rate between two matched methods was calculated to be 82.2% (cluster-corrected 95% CI: 80.4–84.0%). 

The classification performance of panoramic radiographs was tested by converting the data into a binary classification format and comparing it with the reference standard CBCT. The binary diagnostic performance metrics, including sensitivity, specificity, PPV, NPV, accuracy, LR+, and LR−, are presented in Table 3.

Two separate GEE models were developed to address different clinical questions: the first explored factors associated with correct panoramic classification, whereas the second was designed as a clinical prediction model for CBCT-confirmed root protrusion. GEE logistic regression showed that the type of tooth and the CBCT class were important factors that independently predicted the correct classification in panoramic images (Table 4). Although age showed a statistically significant association, the effect size was minimal and is unlikely to be clinically meaningful. Sex was not a significant predictor (*p* = 0.414).

### 3.2. Cluster-Adjusted Clinical Prediction Model

In addition to the diagnostic accuracy model above, a second, separate GEE logistic regression model was developed to predict CBCT-confirmed root protrusion (event = CBCT Class 3, consistent with the outcome definition used in the diagnostic accuracy analysis above; Classes 1–2 as reference) from panoramic classification, tooth type, sex, and age, with the aim of providing a clinically usable prediction tool (Table 5). 

In this model, panoramic Class 2 and Class 3 findings (versus Class 1) were associated with markedly higher odds of true protrusion (OR = 12.80, 95% CI: 5.86–27.99; OR = 110.79, 95% CI: 51.72–237.36, respectively; both *p* < 0.001), as were molar (OR = 18.79) and premolar (OR = 3.60) tooth types compared with canines (both *p* < 0.001). Female sex was associated with lower odds of protrusion (OR = 0.67, 95% CI: 0.50–0.90, *p* = 0.008), and each additional year of age was associated with slightly lower odds of protrusion (OR = 0.97, 95% CI: 0.96–0.98, *p* < 0.001). This model demonstrated excellent discrimination (apparent AUC = 0.948; cluster-based bootstrap 95% CI: 0.941–0.955, 1000 resamples) and satisfactory calibration across risk deciles. The apparent calibration slope was 1.00 (95% CI: 0.92–1.08), and the calibration intercept was 0.00 (95% CI: −0.14 to 0.14); the prevalence of CBCT-confirmed root protrusion was 22.5% (cluster-bootstrap 95% CI: 20.7–24.4%). At a probability threshold of 0.5, the model showed sensitivity = 0.807 and specificity = 0.921; at a threshold matched to the observed prevalence (0.225), sensitivity increased to 0.935 with specificity = 0.848 (full confusion matrices are provided in Supplementary Table S2). To quantify the incremental value of tooth type, age, and sex beyond panoramic classification alone, a reduced model containing panoramic classification only was compared with the full model: apparent AUC increased from 0.916 (panoramic classification only) to 0.948 (full model), a delta AUC of 0.033, with the quasi-likelihood under the independence model criterion (QIC, a GEE analog of the Akaike Information Criterion, where lower values indicate better model fit) improving from 1824.8 to 1626.2. Model optimism was assessed using Harrell’s bootstrap optimism-correction procedure (1000 cluster-based resamples), yielding a mean optimism of 0.0007 and an optimism-corrected AUC of 0.948 (shrinkage factor = 0.999), indicating negligible overfitting. A sensitivity analysis using the five-category tooth-type classification (as in Table 4) in place of the pooled molar/premolar/canine grouping showed marginally better discrimination (AUC = 0.951, QIC = 1584.4) and confirmed that the first premolar category did not differ significantly from the canine reference (OR = 0.57, 95% CI: 0.16–2.02, *p* = 0.381), supporting the parsimonious pooled grouping used in the primary model (Supplementary Table S3). DCA showed that the model provided greater net benefit than treat-all, treat-none, and simple panoramic-classification-based heuristics across the clinically plausible threshold-probability range of approximately 10–50% (Supplementary Figure S1).

**Figure 4:**
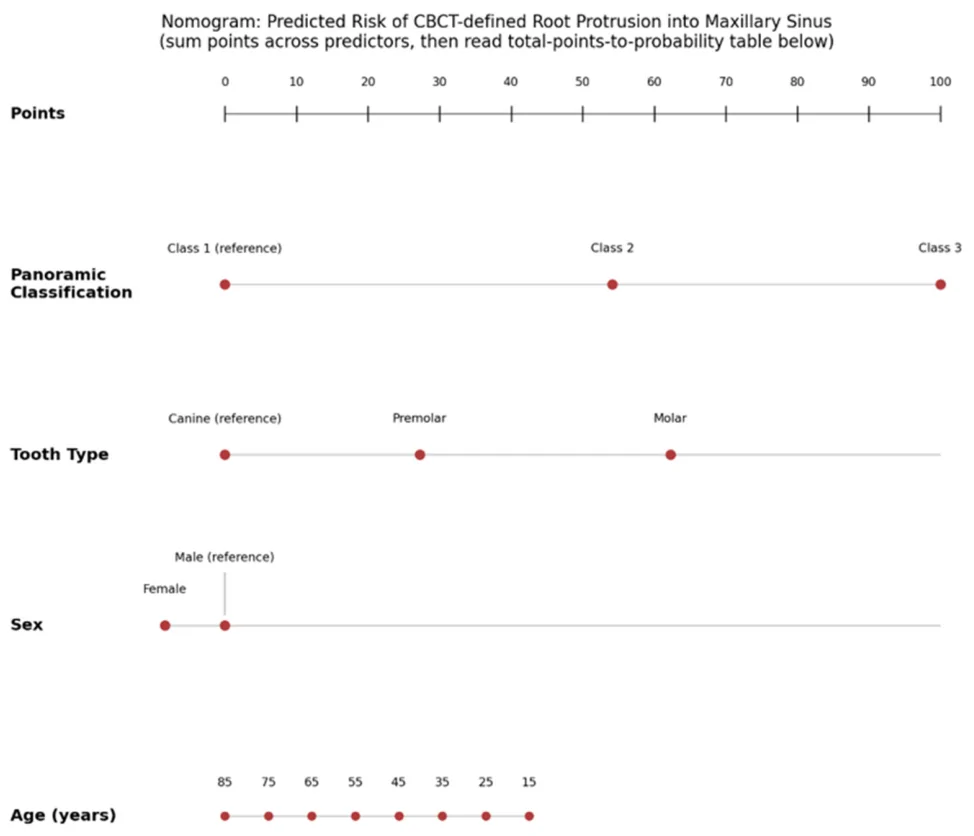
Nomogram derived from the cluster-adjusted GEE logistic regression model for predicting CBCT-confirmed root protrusion, based on panoramic classification, tooth type, sex, and age.

Based on this model, a nomogram and an interactive web-based risk calculator were developed to support clinical decision-making regarding the need for CBCT imaging following an initial panoramic evaluation (Figure 4). A corresponding point-to-probability lookup table is provided as Supplementary Table S1 (Excel), and the interactive calculator is openly available at https://doi.org/10.5281/zenodo.21335638.

The intra-patient correlation coefficient (estimated under an exchangeable study correlation structure) was 0.068. This value indicates a low but non-negligible correlation between teeth from the same patient, supporting the use of the GEE approach. This residual correlation, estimated by the GEE working correlation structure after adjusting for panoramic classification, tooth type, age, and sex, is distinct from the unconditional ICC (0.691) estimated prior to model fitting and used for sample-size planning (Section 2.1); the large reduction from 0.691 to 0.068 indicates that most of the observed clustering was explained by these predictors.

## 4. Discussion

In this study, we evaluated the diagnostic performance of panoramic radiography in detecting the relationships between the maxillary sinus and roots, in comparison to CBCT scans. Our study accounted for within-patient clustering using GEE and cluster-corrected CIs, thereby avoiding artificially narrow CIs that result from treating teeth as independent observations. To our knowledge, this statistical approach has not been widely applied in similar studies [21,22,23,24,25,26,27,35]. When evaluated as a three-category classification, the overall correct classification rate was 82.2%. However, after dichotomization into protrusion and non-protrusion categories, the diagnostic accuracy increased to 87.6%. Although 90% of the roots that were distant from the sinus and 89.9% of the roots that protruded into the sinus were correctly classified using panoramic radiographs, the category in which these radiographs were most misleading involved the roots that showed a close relationship with the sinus floor. Only 40.5% of these cases were correctly classified, while 47.4% were incorrectly classified as being within the sinus. This finding is consistent with previous studies [21,25,27]. 

The high NPV (96.7%) and low LR− (0.118) values of the panoramic imaging method indicate that it is a reliable method for cases where the roots do not protrude into the sinus. However, the PPV (66.6%) and LR+ (6.87), indicating moderate diagnostic values, show that panoramic radiography may produce false-positive findings when evaluating the relationship between the root and the sinus. These values indicate that panoramic radiography has a high probability of predicting the root distant from the sinus; however, it has a moderate ability to predict roots that protrude into it. This finding is supported by the results of other studies [21,25,26,28,35]. Lopes et al. [21] reported an NPV of 89% and a PPV of 70%, while Jung et al. [25] reported NPVs of 97.1% and 97.7% and PPVs of 67.5% and 48.8% for the second and first molars, respectively. Amani et al. [35] stated that if the roots are adjacent to or protruding into the sinus in panoramic images, prescribing CBCT scans should be preferred. 

The first GEE model was designed to identify factors associated with the accuracy of panoramic classification, whereas the second model was intended to provide an individualized prediction tool for clinical decision-making. Analysis of the factors influencing panoramic classification accuracy showed that tooth type and root–sinus relationship type were significant independent predictors of accurate classification in panoramic radiographs. Compared with canines, first and second molars had significantly lower odds of correct classification (OR = 0.188 and 0.196, respectively; both *p* < 0.001). The Class 2 root–sinus relationship was linked to the largest reduction in diagnostic accuracy (OR = 0.172, 95% CI: 0.128–0.229, *p* < 0.001). For each one-year increase in age, the odds of correct panoramic classification increased slightly (OR = 1.011, 95% CI: 1.001–1.021, *p* = 0.029). 

The cluster-adjusted clinical prediction model showed excellent discrimination performance in predicting root protrusion into the maxillary sinus (AUC = 0.948, 95% CI: 0.941–0.955). In this model, as age increases, the probability of protrusion decreases slightly but significantly. Previous studies have demonstrated an age-related increase in root–sinus distance [36,37,38,39]. This observation may be associated with an increase in maxillary sinus volume until approximately age 20, followed by a subsequent decline [40,41]. Moreover, it has been proposed that the physiological eruption of teeth, in response to the reduction in clinical crown height associated with aging, may also contribute to this phenomenon [36]. On the other hand, it has been contended that tooth loss facilitates sinus pneumatization, hence diminishing the distance with age [42]. Our finding of a slight decrease in protrusion probability with increasing age is more consistent with the eruption-based explanation than with the pneumatization-related tooth-loss hypothesis, although the present cross-sectional design cannot establish causality between these mechanisms.

In this study, the probability of a root protrusion into the maxillary sinus was higher in males than in females, possibly reflecting both the greater maxillary sinus volume [43] and the longer tooth roots reported in males [44]. Nonetheless, several studies have indicated no significant difference between sexes regarding relationship patterns [36,38,45]. Variation in these findings may be due to population differences between the studies.

The lower residual intra-patient correlation observed in the GEE model compared with the unconditional ICC (0.068 vs. 0.691, respectively) suggests that much of the clustering was explained by the included predictors, supporting the adequacy of the selected model.

A slight caudocranial inclination of the X-ray beam and non-uniform vertical magnification of the image are important factors that can affect the vertical relationships of anatomical structures in panoramic radiography; these factors may primarily contribute to the tendency to misclassify teeth with “Class 2” root–sinus relationships as “Class 3”. In addition, the superimposition of structures, because it displays three-dimensional structures in two dimensions, and the lack of fine detail are contributory factors to this condition [17]. 

Several studies have investigated panoramic radiographic signs associated with root protrusion into the maxillary sinus [21,24,25,35,46]. ‘Projection of the root apex into the sinus cavity’ [21,24,35], ‘darkening of the involved root apical region’ [24,35], ‘the interruption of the sinus floor’ [21,25], and ‘absence of the periodontal ligament space’ and ‘absence of the lamina dura’ [36] have been suggested as indicative radiographic signs of root protrusion into the sinus. The differences between these findings may be explained by the limited anatomical detail provided by panoramic radiography compared with intraoral periapical radiographs [19]. 

CBCT evaluations revealed that 22.5% of all teeth have roots protruding into the sinus, and 15.7% are in close contact with it. Although some studies have reported the frequency of roots protruding into the sinus to be higher than in our study [21,24], the varied classification types used in the studies may be related to these different findings. Root protrusion into the sinus was most prevalent for the second molars (53.9%) and followed by first molars (49.8%), as found in the previous studies [38,39,45,47]. On the other hand, Tian et al. [40] and Sarilita et al. [48] reported that root protrusion into the sinus was most prevalent for the first molar. In this study, the most common root–sinus relationship for canines and first and second premolars was Class 1. The root protrusion into the sinus was detected in only 1.0% of canines, 0.7% of first premolars, and 13.5% of second premolars. These results are consistent with the previous studies [42,49,50]. The frequency of root protrusion for canines, which was 1.0% in our study, varies between 0.0% and 19.6% in the other studies [23,37,51,52]. In this study, no statistically significant differences were observed between the right and left quadrants of the jaws concerning sinus–root relationships, aligning with those of previous studies [45,53].

CBCT was used as the reference standard due to its superior three-dimensional visualization and high spatial resolution; however, the interpretation of CBCT images can be restricted by various factors such as image resolution and artifacts. Voxel size is the primary factor determining resolution quality; as voxel size increases, resolution decreases. Artifacts including motion, noise, scatter, and beam hardening or streaking significantly limit the interpretation of CBCT imaging. When interpreting CBCT images, these limitations must be considered [17]. 

Because of the higher radiation dose of CBCT compared with conventional panoramic radiography, clinical usage should always be justified according to the ALARA (As Low As Reasonably Achievable) principle. In the evaluation of the relationship between maxillary molar roots and the maxillary sinus, CBCT provides detailed three-dimensional information and may substantially improve diagnostic accuracy in complex anatomical structures. However, CBCT should not be routinely prescribed for every patient as a diagnostic tool. Clinicians should make decisions, especially for young individuals, by considering the balance between the expected diagnostic benefit and the estimated risk [19,20,54]. It is anticipated that the clinical prediction model developed in this study can meet the clinician’s needs in precisely this situation.

The cluster-adjusted clinical prediction model demonstrated that molars showing a “Class 3” panoramic score were associated with a high probability of CBCT-confirmed root protrusion, particularly in younger male patients. In such cases, CBCT may be considered before surgical procedures, using the smallest appropriate FOV in accordance with the ALARA principle. Similarly, molars showing a “Class 2” panoramic score may warrant further three-dimensional evaluation because of the increased likelihood of misclassification. In premolars, the need for CBCT should be determined according to the complexity and extent of the planned procedure. The probability of root protrusion decreases slightly with increasing age; however, this relationship may be modified by tooth loss because of maxillary sinus pneumatization. Sun et al. [27] stated that panoramic radiography tends to overestimate root protrusion and recommended CBCT for suspicious cases. Our findings are consistent with this conclusion but extend the existing evidence by accounting for within-patient clustering using GEE and by providing an individualized prediction model that supports CBCT decision-making rather than relying solely on imaging classification.

Our approach can also be situated within the broader clinical prediction-model literature. The TRIPOD-Cluster guidance formally addresses the reporting of prediction models developed from clustered data, such as repeated observations within participants as in our tooth-within-patient design [55]; our use of GEE-based estimation and cluster-based internal validation aligns with this guidance, although we did not assess heterogeneity across multiple clusters (e.g., centers), since our clustering unit was the patient rather than a center. A recent systematic review of prediction models in dental implantology found that the majority of published dental prediction models show unclear or high risk of bias and that only a minority undergo external validation [56], underscoring that our own model’s developmental, single-center status is a common limitation across dental prediction modeling rather than one unique to this study. A multicenter external validation of a periodontal-prognosis nomogram illustrates both the feasibility and the value of the external validation pathway that our model would need to follow before clinical adoption [57]. 

Certain limitations of this study must be recognized. The data were obtained from a single center, which may have limited representation in the general population. The proximity of the maxillary sinus to the tooth apex is based on the root closest to it. Individually grading each root may be useful in planning surgical procedures for patients. Using the closest root systematically biases classification toward the more severe category for multirooted teeth, which may inflate the reported prevalence of Class 2/3 relationships relative to a per-root or average-root approach; per-root analysis is an important direction for future work. In addition, since there are no studies directly addressing clustering at the tooth level in terms of sinus–root relationships, a conservative estimate from the general dentistry clustering literature was used. However, the observed ICC in the final dataset was higher, reflecting stronger within-patient clustering than initially anticipated. Patients who underwent both panoramic imaging and CBCT are unlikely to represent the average dental population, since CBCT is typically prescribed for more complex cases; this introduces potential referral and spectrum bias, which may overestimate diagnostic performance relative to routine, low-risk dental practice, and limits generalizability to unselected screening populations. 

Panoramic radiographs were acquired using two different devices with different exposure parameters, and CBCT was acquired using a single device; the device of acquisition was not retrievable at the patient level in this retrospective dataset, so potential effects of inter-device variation on classification accuracy could not be evaluated. Reliability of the CBCT reference standard itself was not formally assessed via repeat readings; the two-observer consensus process with third-observer adjudication for disagreements was assumed sufficient but was not empirically validated with kappa statistics. The two observers who evaluated the panoramic radiographs were the same observers who evaluated the CBCT reference standard; although panoramic evaluation was deferred two weeks after CBCT assessment to reduce recall, this constitutes a time-separated evaluation by the same individuals rather than a formal blinded evaluation with independent observers.

Finally, the specific reasons for exclusion among the 6543 records not meeting eligibility criteria were not individually logged during the retrospective screening process, precluding a fully itemized breakdown in the STARD flow diagram (Figure 3) and limiting formal assessment of selection bias at this stage. Future prospective studies should address these points by recording the device of acquisition, formally assessing CBCT reference-standard reliability, using independent blind observers, and logging exclusion reasons individually. External validation of a clinical prediction model in an independent population is required before routine clinical implementation.

## 5. Conclusions

Maxillary molar roots frequently protrude into the sinus; this possibility should be considered during dental procedures performed on these teeth. While panoramic radiography is a reliable diagnostic tool for detecting roots that are a distance from the sinus, the method may overestimate the proximity when roots are close to the sinus floor due to false-positive results. Therefore, when deciding which imaging modality should be prescribed for examination of the root–sinus relationship, the scope of the planned procedure and the patient’s specific needs should be considered. The cluster-adjusted clinical prediction model, developed and evaluated internally in this single-center cohort and considering the patient’s sex, age, and tooth type, has the potential to support clinicians in selecting patients for CBCT imaging following successful external validation; it should not yet be recommended for routine clinical use. Pending such validation, it may help clinicians weigh anticipated diagnostic benefit against radiation exposure when forecasting the outcome of a proposed procedure.

## Figures and Tables

**Table 1:** Distribution of the types of root apex–sinus relationships by tooth type (%).

	Root-Sinus Relationship Classification			
	1	2	3	Total
Canine	751 (96.8)	17 (2.2)	8 (1.0)	776 (100)
1st premolar	682 (93.8)	40 (5.5)	5 (0.7)	727 (100)
2nd premolar	395 (61.8)	158 (24.7)	86 (13.5)	639 (100)
1st molar	144 (24.9)	146 (25.3)	288 (49.8)	578 (100)
2nd molar	153 (21.4)	177 (24.7)	386 (53.9)	716 (100)
Total	2125 (61.8)	538 (15.7)	773 (22.5)	3436 (100)

**Table 2:** Contingency table presenting the root–sinus relationship according to CBCT and panoramic imaging methods (%).

Imaging Methods and Scores		CBCT			Total
		1	2	3	
**Panoramic**	1	1912 (90)	65 (12.1)	10 (1.3)	1987 (57.8)
	2	120 (5.6)	218 (40.5)	69 (8.9)	407 (11.8)
	3	93 (4.4)	255 (47.4)	694 (89.8)	1042 (30.3)
Total		2125 (100)	538 (100)	773 (100)	3436 (100)

**Table 3:** Diagnostic performance of panoramic radiography for detecting maxillary sinus root protrusion.

Performance Metric	Value	95% CI
Sensitivity	0.898	0.875–0.920
Specificity	0.869	0.855–0.884
PPV	0.666	0.632–0.697
NPV	0.967	0.959–0.975
LR+	6.87	6.14–7.71
LR−	0.118	0.092–0.145
Accuracy	0.876	0.864–0.887

Positive class: root protruding into sinus (CBCT Class 3); negative class: root outside sinus (CBCT Classes 1 + 2). Test positive: panoramic Class 3. Point estimates are unaffected by clustering; 95% CIs were cluster-corrected using patient-level bootstrapping (*n* = 1000 iterations).

**Table 4:** Factors associated with correct panoramic classification.

Variable	OR	95% CI	*p*
*Tooth type*			
First premolar	0.781	0.555–1.101	0.159
Second premolar	0.275	0.191–0.397	<0.001
First molar	0.188	0.120–0.296	<0.001
Second molar	0.196	0.126–0.305	<0.001
*CBCT class*			
Class 2	0.172	0.128–0.229	<0.001
Class 3	2.994	1.989–4.505	<0.001
*Continuous variables*			
Age	1.011	1.001–1.021	0.029
Sex (male vs. female)	1.110	0.864–1.427	0.414

The dependent variable was correct classification (1 = correct, 0 = incorrect). Reference categories: ‘canine’ for tooth type, ‘Class 1’ for CBCT class, ‘female’ for sex, and age (per 1-year increase).

**Table 5:** Cluster-adjusted GEE logistic regression model for CBCT-confirmed root protrusion.

Variable	OR	95% CI	*p*
Panoramic class 2 (vs. 1)	12.80	5.86–27.99	<0.001
Panoramic class 3 (vs. 1)	110.79	51.72–237.36	<0.001
Tooth type: Molar (vs. canine)	18.79	7.82–45.13	<0.001
Tooth type: Premolar (vs. canine)	3.60	1.53–8.49	0.003
Sex: Female (vs. male)	0.67	0.50–0.90	0.008
Age	0.97	0.96–0.98	<0.001

CBCT Class 3 vs. Classes 1–2, with panoramic classification, tooth type, sex, and age (per year) as predictors.

## Data Availability

The raw data supporting the conclusions of this article will be made available by the authors on request. The GEE logistic regression code, the point-to-probability lookup table generation script, and the interactive risk calculator source code are openly available in a version-controlled Zenodo repository (https://doi.org/10.5281/zenodo.21335638). All statistical outputs, model coefficients, and calculator logic were independently reviewed and verified by the authors against the reported results before use.

## Supplementary Materials

The following supporting information can be downloaded at: https://www.mdpi.com/article/10.3390/diagnostics16162580/s1, Supplementary Table S1: Point-to-probability lookup table for the clinical prediction model (Excel); Supplementary Figure S1: Decision curve analysis comparing the prediction model against treat-all, treat-none, and panoramic-classification-based heuristics; Supplementary Table S2: Prevalence, apparent calibration slope/intercept, and confusion matrices at a 0.5 threshold and a prevalence-matched threshold; Supplementary Table S3: Sensitivity analysis of the prediction model using the five-category tooth-type classification.

## Abbreviations

The following abbreviations are used in this manuscript:

AUC	Area Under the Curve
CBCT	Cone-Beam Computed Tomography
CI	Confidence Interval
CIs	Confidence Intervals
CT	Computed Tomography
DEFF	Design Effect
FOV	Field of View
GEE	Generalized Estimating Equations
ICC	Intraclass Correlation Coefficient
LR+	Positive Likelihood Ratio
LR−	Negative Likelihood Ratio
NPV	Negative Predictive Value
PPV	Positive Predictive Value
QIC	Quasi-Likelihood Under the Independence Model Criterion
