# Supplementary material for: Diagnostic Accuracy of Panoramic Radiography for Assessing Maxillary Posterior Root Protrusion into the Maxillary Sinus: A Cluster-Adjusted Prediction Model Using CBCT as Reference
Source: Diagnostics (Basel). 2026 Aug 15;16(16):2580. doi: 10.3390/diagnostics16162580 (PMC13512893; doi:10.3390/diagnostics16162580)
Supplement: Supplementary file 1 [file diagnostics-16-02580-s001.zip › Supplementary_Table_S2.pdf]

Supplementary Table S2. Prevalence, apparent calibration, and confusion matrices for the cluster-adjusted clinical prediction model.

Prevalence of CBCT-confirmed root protrusion (Class 3) was 22.5% (cluster-bootstrap 95% CI: 20.7–24.4%). The apparent calibration slope was 1.00 (95% CI: 0.92–1.08) and the apparent calibration intercept was -0.00 (95% CI: -0.14 to 0.14). These are apparent (in-sample, non-externally-validated) estimates and should be interpreted accordingly.

Threshold	TN, n (%)	FP, n (%)	FN, n (%)	TP, n (%)	Sensitivity	Specificity	PPV	NPV
0.5	2452 (71.4%)	211 (6.1%)	149 (4.3%)	624 (18.2%)	0.807	0.921	0.747	0.943
Prevalence-matched (0.225)	2259 (65.7%)	404 (11.8%)	50 (1.5%)	723 (21.0%)	0.935	0.848	0.642	0.978

TN: true negative; FP: false positive; FN: false negative; TP: true positive; PPV: positive predictive value; NPV: negative predictive value. Confusion matrices are shown at a fixed 0.5 probability threshold and at a threshold matched to the observed prevalence (0.225), illustrating the sensitivity/specificity trade-off across operating points.
