# Supplementary material for: Diagnostic Accuracy of Panoramic Radiography for Assessing Maxillary Posterior Root Protrusion into the Maxillary Sinus: A Cluster-Adjusted Prediction Model Using CBCT as Reference
Source: Diagnostics (Basel). 2026 Aug 15;16(16):2580. doi: 10.3390/diagnostics16162580 (PMC13512893; doi:10.3390/diagnostics16162580)
Supplement: Supplementary file 1 [file diagnostics-16-02580-s001.zip › Supplementary_Table_S3.pdf]

Supplementary Table S3. Sensitivity analysis: cluster-adjusted GEE prediction model using the five-category tooth-type classification.

Model discrimination and fit: pooled model (Canine/Premolar/Molar, as reported in manuscript Table 5) apparent AUC = 0.948, QIC = 1626.2; five-category model apparent AUC = 0.951, QIC = 1584.4. The five-category model shows marginally better discrimination and fit, but the first premolar category does not differ significantly from the canine reference, supporting the pragmatic pooled grouping used in the primary prediction model (manuscript Table 5).

Term	OR	95% CI	p-value
Intercept	0.006	—	<0.001
Panoramic Class 2 (vs 1)	9.03	4.21–19.36	<0.001
Panoramic Class 3 (vs 1)	87.15	41.34–183.72	<0.001
Tooth type: First premolar (vs Canine)	*0.57*	*0.16–2.02*	*0.381*
Tooth type: Second premolar (vs Canine)	5.46	2.21–13.52	<0.001
Tooth type: First molar (vs Canine)	15.41	6.15–38.56	<0.001
Tooth type: Second molar (vs Canine)	26.59	10.69–66.14	<0.001
Sex: Female (vs Male)	0.68	0.50–0.92	0.012
Age (per year)	0.97	0.96–0.98	<0.001

OR: odds ratio; CI: confidence interval (cluster-robust, GEE-based). Reference categories: Panoramic Class 1, Canine, Male. Model fitted using GEE logistic regression with an exchangeable working correlation structure, clustered by patient (n = 484 patients, 3436 teeth). Italicized row (First premolar) indicates a non-significant effect relative to the canine reference.
